# Multiple infliximab biosimilar switches appear to be safe and effective in a real‐world inflammatory bowel disease cohort

**DOI:** 10.1002/ueg2.12357

**Published:** 2023-02-17

**Authors:** Beatriz Gros, Nikolas Plevris, Nathan Constantine‐Cooke, Mathew Lyons, Claire O'Hare, Colin Noble, Ian D. Arnott, Gareth‐Rhys Jones, Charlie W. Lees, Lauranne A. A. P. Derikx

**Affiliations:** ^1^ Edinburgh IBD Unit Western General Hospital Edinburgh UK; ^2^ Department of Gastroenterology and Hepatology Reina Sofía University Hospital Córdoba Spain; ^3^ MRC Human Genetics Unit Institute of Genetics and Cancer University of Edinburgh Western General Hospital Edinburgh UK; ^4^ Centre for Genomics and Experimental Medicine Institute of Genetics and Cancer University of Edinburgh Western General Hospital Edinburgh UK; ^5^ Edinburgh Pharmacy Unit Western General Hospital Edinburgh UK; ^6^ Centre for Inflammation Research The Queen's Medical Research Institute University of Edinburgh Edinburgh UK; ^7^ Department of Gastroenterology and Hepatology Inflammatory Bowel Disease Center Radboud University Medical Center Nijmegen The Netherlands; ^8^ Department of Gastroenterology and Hepatology Erasmus MC, University Medical Centre Rotterdam Rotterdam The Netherlands

**Keywords:** biosimilar, inflammatory bowel disease, infliximab, real world evidence

## Abstract

**Background:**

Switching from originator infliximab (IFX) to biosimilar IFX is effective and safe. However, data on multiple switching are scarce. The Edinburgh inflammatory bowel disease (IBD) unit has undertaken three switch programmes: (1) Remicade to CT‐P13 (2016), (2) CT‐P13 to SB2 (2020), and (3) SB2 to CT‐P13 (2021).

**Objective:**

The primary endpoint of this study was to assess CT‐P13 persistence following switch from SB2. Secondary endpoints included persistence stratified by the number of biosimilar switches (single, double and triple), effectiveness and safety.

**Methods:**

We performed a prospective, observational, cohort study. All adult IBD patients on IFX biosimilar SB2 underwent an elective switch to CT‐P13. Patients were reviewed in a virtual biologic clinic with protocol driven collection of clinical disease activity, C‐reactive protein (CRP), faecal calprotectin (FC), IFX trough/antibody levels, and drug survival.

**Results:**

297 patients (CD *n* = 196 [66%], ulcerative colitis/inflammatory bowel disease unclassified *n* = 101, [34%]) were switched (followed‐up: 7.5 months [6.8–8.1]). This was the third, second and first IFX switch for 67/297 (22.5%), 138/297 (46.5%) and 92/297 (31%) of the cohort respectively. 90.6% of patients remained on IFX during follow‐up. The number of switches was not independently associated with IFX persistence after adjusting for confounders. Clinical (*p* = 0.77), biochemical (CRP ≤5 mg/ml; *p* = 0.75) and faecal biomarker (FC<250 µg/g; *p* = 0.63) remission were comparable at baseline, week 12 and week 24.

**Conclusion:**

Multiple successive switches from IFX originator to biosimilars are effective and safe in patients with IBD, irrespective of the number of IFX switches.

## Graphical abstract

Clinical, biochemical and faecal biomarker remission were comparable at baseline, week 12 and week 24. Number of switches was not independently associated with persistence after adjusting for confounders.

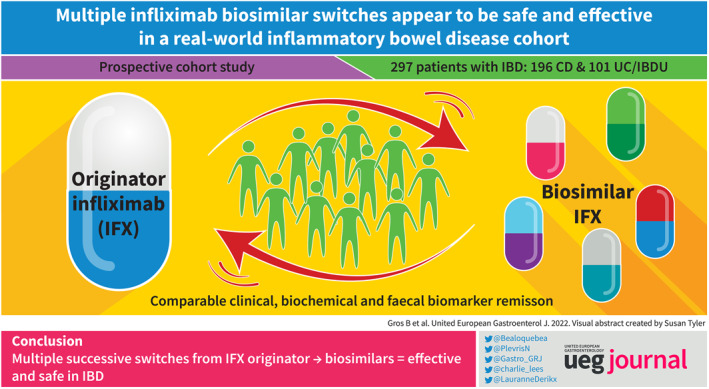

**Key Summary**

**Established knowledge on this subject:**

• Data on single infliximab biosimilar switch has been proved to be effective and safe.
• Multiple biosimilars are available and data on multiple successive switches are scarce.

**New findings:**

• In our study we observed similar effectiveness, safety and immunogenicity rates across different number of biosimilar infliximab switches (single, double and triple).
• These results will help making decisions on multiple switches as more and more biosimilars are becoming available and therefore will help saving costs.

## INTRODUCTION

Infliximab (IFX), a monoclonal antibody inhibiting anti‐tumour necrosis factor (TNF), is a widely used biologic therapy whose originator (Remicade®) was the first biologic drug approved for the treatment of inflammatory bowel disease (IBD) in 1998.^1^

The first biosimilar of IFX approved by the US Food and Drug Administration and the European Medicines Agency was CTP‐13 in 2013.^2^ This approval was based on data extrapolated initially from the PLANETRA and PLANETAS studies in rheumatoid arthritis and ankylosing spondylitis.^3^, ^4^ Safety and efficacy data in IBD were provided by the NOR‐SWITCH^5^ and CT‐P13 3.4 randomised control trials.^6^ This was reflected in an updated ECCO position stating that switching from IFX originator to a biosimilar is acceptable with evidence for safety and efficacy.^7^

The reason for the development of biosimilars was mainly economic.^8^, ^9^ Reducing costs has increased IFX availability worldwide with many patients benefiting from it as growing evidence proves early therapy is associated to better outcomes.^10^, ^11^

Single and double switch appeared to be effective and safe in some observational studies, but data regarding three or more switches are lacking.^12^, ^13^ In the present economic climate with multiple biosimilars available at competitive prices, data about multiple biosimilar switches is of increasing importance.

In the Edinburgh IBD Unit, we previously implemented a managed switch programme guiding the transition from IFX originator to the IFX biosimilar CT‐P13 which appeared to be safe and effective.^14^ Given a further price reductions of IFX biosimilars following successive annual rounds of tendering by National Procurement Scotland, similar switch programmes were implemented in q1 2020 (SB2) and q4 2021 (CT‐P13). In the present study we aimed to assess effectiveness and safety of this third IFX biosimilar switch (SB2 to CT‐P13) including patients who have had a single, double or triple switch.

## METHODS

### Study design and outcomes

We performed a prospective observational cohort study in the Edinburgh IBD unit, a tertiary IBD referral centre in NHS Lothian (Scotland), to investigate the effectiveness and safety of the third IFX biosimilar switch (SB2 to CT‐P13) including patients who have had single, double and triple switches. NHS Lothian provides universal, free at point of care healthcare for a population of 912,490 people (estimate mid 2020), including more than 8000 patients with IBD.^15^

Three managed switch programmes for IFX have been undertaken in the Edinburgh IBD unit, including: (1) IFX originator to CT‐P13 in 2016,^14^ (2) CT‐P13 to SB2 (March–May 2020), and (3) SB2 to CT‐P13 in 2021 (6 October 2021–30 November 2021). All adult IBD patients underwent an elective biosimilar switch in these programmes, regardless of IBD phenotype, disease activity and IFX dosing. They received correspondence from the unit informing them of the planned biosimilar switch and that this process would be closely monitored as part of their routine care. Dosing and interval remained unchanged following the switch unless clinical need or subtherapeutic drug levels dictated therapy adjustments. Patients were regularly reviewed in a virtual biologic clinic as part of routine clinical care. At this time, clinical disease activity scores (Harvey Bradshaw Index, HBI; partial Mayo score), laboratory parameters (including C‐reactive protein [CRP], IFX trough and antibody levels) and faecal calprotectin (FC) were collected.

### Patients

We identified all adult IBD patients receiving IFX biosimilar SB2 in the Edinburgh IBD unit from our infusion suite records who underwent an elective switch to CT‐P13 (6 October 2021–30 November 2021). Patients who received three doses of SB2 (and thus completed IFX induction therapy) were eligible for inclusion. Exclusion criteria included microscopic colitis and checkpoint inhibitor colitis.

### Outcomes

The primary endpoint of this study was CT‐P13 drug persistence. Secondary endpoints included clinical remission (CD: Harvey Bradshaw Index (HBI) <5; UC: partial Mayo <2),^16^ biochemical remission (CRP ≤5 mg/L) and faecal biomarker remission (FC ≤250 µg/g)^17^, ^18^ at baseline (biosimilar switch), week 12 (±4 weeks) and week 24 (±4 weeks). Subgroup analysis for both drug persistence and effectiveness were performed based on the number of IFX switches. In addition we assessed immunogenicity parameters (IFX drug and antibody levels) and safety parameters (adverse events).

### Data collection

Patient demographics and IBD characteristics were extracted from electronic medical health records. We collected the following data: sex, smoking history, body mass index, IBD type, age at IBD diagnosis, disease extent and behaviour according to the Montreal classification and both previous and ongoing exposure to IBD‐related medical therapies. IFX start date, dose, dose interval and previously used IFX brands were recorded.

We prospectively collected clinical scores (HBI and partial Mayo), CRP, FC and IFX drug and antibody levels at baseline, week 12 (±4 weeks) and week 24 (±4 weeks). Data were collected at the infusion suite prior to infusion of IFX. If no recent value of calprotectin was available within the 4 weeks prior to switch, patients were given a stool sample kit at the infusion suite to submit at their GP the same week. Given different IFX intervals, a 4‐week time margin was used whilst collecting follow‐up data. Furthermore, we recorded IFX dose adjustments as well as IFX stop dates and reason for treatment discontinuation.

Primary non‐response was defined as lack of clinical and biochemical response in the first 4 months since IFX was started requiring treatment discontinuation. Secondary loss of response was defined as clinical and biochemical relapse in patients who previously responded. In patients with no detectable IFX trough levels and detectable antibody levels who discontinued IFX, we considered immunogenicity as the reason for treatment discontinuation. Thus, secondary loss of response was considered in the absence of immunogenicity.

All adverse events during follow‐up were documented. A serious adverse event was defined as an adverse event leading to IFX suspension or discontinuation, hospitalisation, or death. Adverse events occurring in <5 patients are reported as ‘<5 events’ to avoid the use of personally identifiable information which can be traced back to a person.

### Faecal calprotectin

All FC were measured in the Western General Hospital, Edinburgh, with a standard enzyme‐linked immunosorbent assay (ELISA) technique (Calpro AS™) resulting in numerical values between 20 and 1250 μg/g.

### Infliximab drug levels and antibody assay

Since January 2018, IFX trough levels have been analysed at the Queen Elizabeth University Hospital, Glasgow, using Immundiagnostik monitor ELISA as per the manufacturer's protocol. The lower and upper limits are respectively <0.3 and >14.4 μg/ml for IFX through levels and ×10 and 400 AU/L for IFX antibodies. Drug tolerant anti‐drug antibody assays are used and antibody testing is only performed when through levels are below 7 μg/ml or when IFX antibodies have previously been detectable.

### Statistics

All analyses were performed with IBM SPSS statistical software package version 25. We used descriptive statistics to describe baseline characteristics. Continuous variables are expressed as medians and interquartile range or mean and standard deviation, depending on distribution and were analysed with a Student *t*‐test or Mann‐Whitney *U* test as appropriate. Categorical variables were reported as frequencies and were analysed with chi‐square/Fisher's exact test.

Kaplan‐Meier curves were calculated for drug survival. Time‐to‐event was calculated from IFX switch until discontinuation of IFX biosimilar CT‐P13. Patients were censored at the end of follow‐up, which was defined as the last data collection point. We performed explorative analyses with univariable and multivariable Cox regression analyses to identify factors independently associated with drug survival. In case of a *p*‐value of <0.1 in univariable analysis, variables were included in the multivariable analysis. A *p*‐value of <0.05 was considered statistically significant.

Clinical, biochemical and faecal biomarker remission were analysed as categorical variables. We performed an intention‐to‐treat analysis with the last observation carried forward. In addition, we performed a sensitivity intention‐to‐treat analysis with only the last observation carried forward for patients who discontinued IFX, not considering patients with missing data, to provide a conservative estimate of remission. Comparison of parameters at the three different time points (baseline, 12 and 24 weeks) was done using Freidman analysis and if significant pairwise comparison at each time point was carried out using Wilcoxon signed rank test.

### Ethics

This work was considered a service evaluation/audit as all data were collected as part of routine clinical care. Therefore, no written consent or formal ethical approval was necessary as per departmental policy and Health Research Authority.^19^ This piece of work conforms to the ethical guidelines of the 1975 Declaration of Helsinki as reflected in a prior approval by the institution's review board.

## RESULTS

### Study population

A total of 297 patients (CD *n* = 196 [66%], ulcerative colitis [UC]/inflammatory bowel disease unclassified [IBDU] *n* = 101 [34%]) were switched from SB2 to CT‐P13 and followed up for a median of 7.5 months (6.8–8.1) (Table 1). 183 patients (61.6%) were male with a median IBD duration of 6.4 years (2.4–11.4). Most CD patients had ileocolonic (L3) disease distribution (84/195, 43.1%), and 62/196 patients (31.8%) had perianal disease activity. Of 89 UC patients, 45 (50.6%) had extensive/pancolitis (E3).

**TABLE 1:** Baseline characteristics of inflammatory bowel disease patients on infliximab at switch from SB2 to CT‐P13

Variable	Total cohort (*N* = 297)	Patients who had 3 switches (*n* = 67)	Patients who had 2 switches (*n* = 138)	Patients who had 1 switch (*n* = 92)	*p*‐ value
Sex, male, *n* (%)	183 (61.6)	45 (67.2)	83 (60.1)	55 (59.8)	0.57
Current age, median (IQR)	37 (29–51.5)	40 (32–56)	37 (28.8–52.3)	35 (28–45.8)	0.08
BMI, kg/m^2^, median (IQR)	26.7 (23.7–30.3)	26.3 (24.5–31.1)	27.4 (23.8–30.7)	26 (23.1–29)	0.34
Disease duration, years, median (IQR)	6.4 (2.4–11.4)	11.4 (9.4–18.4)	6.3 (3.4–11.4)	2.3 (0.4–7.4)	**<0.0001**
Disease type					
–Crohn's disease, *n* (%)	196 (66)	61 (91)	86 (62.3)	49 (53.3)	**<0.0001**
–Ulcerative colitis/IBDU, *n* (%)	101 (34)	6 (9)	52 (37.7)	43 (46.7)	
Ulcerative colitis Montreal classification, *n* (%)					
–E1	12 (4)	0	7 (5.1)	5 (5.4)	0.09
–E2	32 (10.8)	0	17 (12.3)	15 (16.3)	
–E3	45 (15.2)	5 (7.5)	21 (15.2)	19 (20.7)	
Crohn's disease location, *n* (%)					
–Ileal (Montreal L1)	34 (11.4)	6 (9)	16 (11.6)	12 (13)	0.19
–Colonic (Montreal L2)	77 (25.9)	27 (40.3)	30 (21.7)	20 (21.7)	
–Ileocolonic (Montreal L3)	84 (28.3)	29 (43.3)	38 (27.5)	17 (18.5)	
–Upper GI (Montreal L4)	27 (9.1)	11 (16.4)	12 (8.7)	4 (4.3)	0.35
–Perianal disease	64 (21.5)	23 (34.3)	28 (20.3)	13 (14.1)	**0.01**
Crohn's disease behaviour, *n* (%)					
–Non‐stricturing, non‐penetrating (Montreal B1)	143 (48.1)	45 (67.2)	60 (43.5)	38 (41.3)	0.8
–Stricturing (Montreal B2)	27 (9.1)	11 (16.4)	12 (8.7)	2 (4.2)	
–Penetrating (Montreal B3)	25 (8.4)	6 (9)	12 (8.7)	7 (7.6)^a^	
Extraintestinal manifestation, *n* (%)	85 (28.9)	23 (34.3)	41 (29.7)	21 (22.8)	0.27
Time on infliximab, years, median (IQR)	3.1 (1.3–5.1)	8.6 (6.7–10.5)	3.2 (2.6–4.1)	0.6 (0.3–1.2)	**<0.0001**
Previous biologic/small molecules, *n* (%)	32 (10.8)	1 (1.5)	8 (5.8)	23 (25)	**<0.0001**
–AntiTNF	25 (8.4)	0	8 (5.8)	17 (18.5)	**<0.0001**
–Vedolizumab	7 (2.4)	0	1 (0.7)	6 (6.6)	**0.002**
–Tofacitinib	7 (2.4)	1 (1.5)	0	6 (6.6)	**0.006**
–Ustekinumab	4 (1.4)	0	1 (0.7)	3 (3.3)	0.154
Concomitant therapy					
–Immunosuppressant, *n* (%)	163 (54.9)	12 (17.9)	69 (50.1)	82 (89.1)	**<0.0001**
–Corticosteroids, *n* (%)	11 (3.7)	1 (1.5)	3 (2.2)	7 (7.6)	0.089
IFX dose and interval at switch, *n* (%)					
–5 mg/kg q8w	167 (56.2)	25 (37.3)	68 (49.3)	74 (80.4)	**<0.0001**
–5 mg/kg q6w	53 (17.8)	24 (35.8)	24 (17.4)	5 (5.4)	**<0.0001**
–5 mg/kg q4w	10 (3.4)	1 (1.5)	5 (3.6)	4 (4.3)	0.60
–10 mg/kg q8w	46 (15.5)	10 (14.9)	30 (21.7)	6 (6.5)	**0.008**
–10 mg/kg q6w	18 (6.4)	6 (9)	9 (7.2)	3 (3.3)	0.32
–10 mg/kg q4w	2	0	2 (1.4)	0	0.32
–Other (5 mg/kg q10w)	1 (0.3)	1 (1.5)	0	0	

*Note*: Bold values highlight statistically significant differences.

Abbreviations: CD, Crohn’s disease; CRP, C–reactive protein; FC, faecal calprotectin; IBDU, Inflammatory bowel disease unclassified; IFX, infliximab; UC, Ulcerative colitis.

^a^ Two missing data.

This switch from SB2 to CT‐P13 was the third successive IFX biosimilar switch for 67/297 patients (22.5%; previous treatment with IFX originator, CT‐P13 and SB2 before switch to CT‐P13), the second for 138/297 patients (46.5%; previous treatment with CT‐P13 and SB2), and the first for 92/297 patients (31%; previous treatment with SB2). Patients were treated with IFX for median 3.1 years (1.3–5.1) prior to current switch. Of 297 patients, 265 (89.2%) were biologic and small molecule naïve. Patients who underwent multiple IFX biosimilar switches had more often Crohn's disease (*p* = 0.0001) with perianal disease activity (*p* = 0.01), a significantly longer disease (*p* = 0.0001) and IFX duration (*p* = 0.0001), were less often on combination therapy with an immunomodulator (*p* = 0.0001), and were more frequently biologic‐naïve prior to IFX (*p* = 0.0001) (Table 1).

### IFX persistence

**FIGURE 1:**
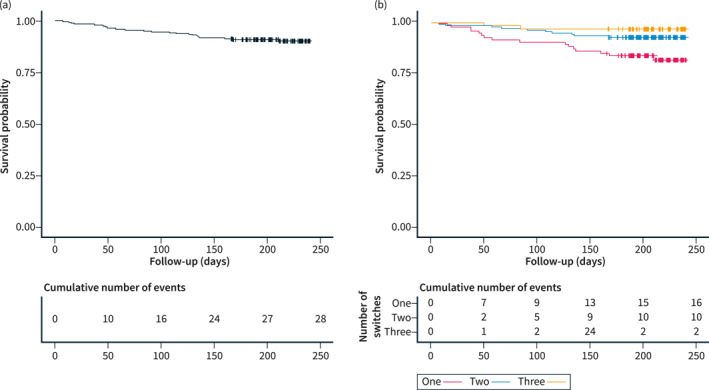
Kaplan‐Meier curves for infliximab treatment persistence. (a) Survival across the total cohort. (b) Survival stratified by number of total infliximab switches. The start of follow‐up is defined as the time of switch.

Of 297 patients, 28 (9.4%) patients discontinued IFX treatment during follow‐up. Median time to IFX discontinuation was 18.5 months (9–48) from IFX commencing and 2.8 months (1.5–4.5) from switch. Reasons for IFX discontinuation included immunogenicity (15/297; 5.1%), secondary loss of response (7/297, 2.4%), adverse events (3/297, 1%), patient's choice (2/297, 0.7%), and primary non‐response (1/297, 0.3%). Of 297 patients, 269 (90.6%) remained on IFX at week 24 (Figure 1).

Subgroup analysis based on number of IFX switches revealed that IFX persistence was 82.6%, 92.8% and 97% in patients with in respective one, two and three IFX switches (*p* = 0.003). However the number of switches was not independently associated with IFX persistence after adjusting for confounders (Table 2). Multivariable analysis identified absence of biochemical remission (CRP >5 mg/L (hazard ratio [HR] 3.21; 95% confidence interval [CI] 1.43–7.24); a diagnosis of UC/IBDU (HR 2.69; 95% CI 1.19–6.06), detectable antibodies against IFX at switch (HR 5.81; 95% CI 2.27–12.84) and time on IFX (HR 0.77; 95% CI 0.62–0.95) as independent predictors for IFX persistence rather than number of IFX switches.

**TABLE 2:** Variables associated with infliximab persistence

Variable	Univariable Cox regression			Multivariable Cox regression^a^		
	Hazard ratio	95% CI	*p*	Hazard ratio	95% CI	*p*
Disease duration	0.79	0.56–1.11	0.17			
Duration of IFX treatment	0.514	0.35–0.76	0.001	0.77	0.62–0.95	0.015
UC/IBDU vs. CD	0.32	0.15–0.68	0.003	2.69	1.19–6.06	0.018
Perianal disease	1.61	0.56–4.66	0.38			
Biologic/small molecule naïve	0.38	0.15–0.94	0.037			
Number of switches	0.40	0.22–0.73	0.003			
Clinical remission at switch	1.34	0.57–3.15	0.50			
CRP >5 mg/L at switch	2.96	1.34–6.54	0.007	3.21	1.43–7.24	0.005
FC ≥250 μg/gr at switch	1.57	0.52–4.69	0.40			
IFX antibodies at switch	5.44	2.47–11.99	<0.0001	5.81	2.63–12.84	<0.0001

Abbreviations: CD, Crohn's disease; CRP, C‐reactive protein; FC, faecal calprotectin; IBDU, inflammatory bowel disease unclassified; IFX, infliximab; UC, ulcerative colitis.

^a^ The final model of the multivariable model is shown here. The following variables were included in the initial multivariable Cox regression model: duration of infliximab treatment, ulcerative colitis versus. Crohn's disease, biologic or small molecule naïve, number of switches, CRP > 5 mg/L at switch and infliximab antibodies at switch.

### Effectiveness

**FIGURE 2:**
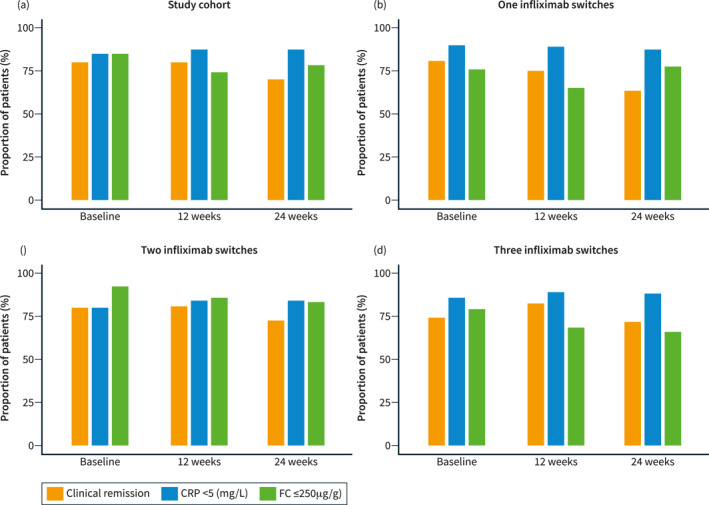
Clinical, biochemical and faecal biomarker remission compared at baseline, week 12 and week 24. Last observation carried forward was used only for those who stopped infliximab during follow‐up. (a) Total cohort: clinical (*p* = 0.005 [baseline vs. 24 weeks, *p* = 0.004]), biochemical (*p* = 0.18) and faecal biomarker (*p* = 0.14) remission rates. (b) Single switch group: clinical (*p* = 0.003 [baseline versus 24 weeks, *p* = 0.034 and 12 week versus 24 weeks, *p* = 0.008]), biochemical (*p* = 0.85), faecal biomarker (*p* = 0.16) remission rates. (c) Double switch group: clinical (*p* = 0.70), biochemical (*p* = 0.26), faecal biomarker (*p* = 0.039 [baseline vs. 12 weeks, *p* = 0.046; baseline vs. 24 weeks, *p* = 0.046]) remission rates. (d) Triple switch group: clinical (*p* = 0.63), biochemical (*p* = 0.67), faecal biomarker (*p* = 0.61) remission rates. CRP, C‐reactive protein; FC, faecal calprotectin.

**FIGURE 3:**
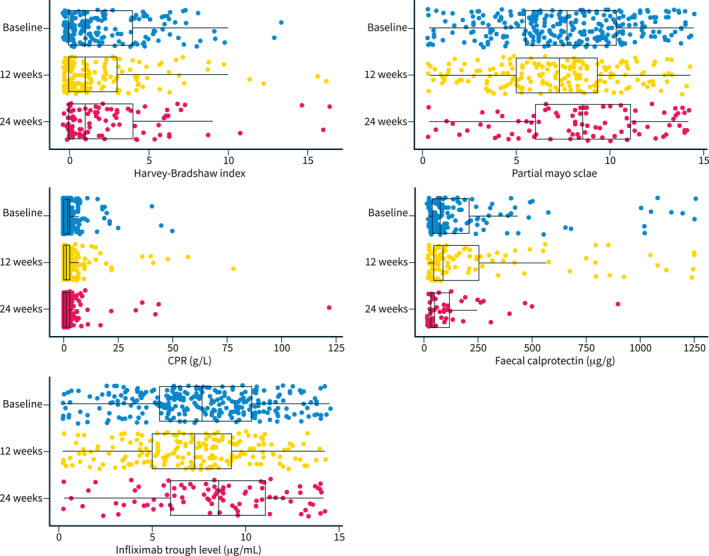
Harvey‐Bradshaw Index, partial Mayo score, CRP, faecal calprotectin and infliximab trough levels at baseline and at 12 and 24 weeks after switch.

Clinical (*p* = 0.77), biochemical (*p* = 0.75) and faecal biomarker (*p* = 0.63) remission rates were comparable at baseline, week 12 and week 24. At baseline, 79.4%, 85.2% and 85.3% were in clinical, biochemical and faecal biomarker remission respectively, versus 81%, 86.5% and 84.4% at week 24. Our sensitivity analysis providing a conservative estimate of remission demonstrated a lower clinical remission rate at week 24 (79%–4% vs. 20.5%; *p* = 0.004) (Figure 2). Median HBI (baseline: 1; week 24: 1), partial Mayo (baseline: 1; week 24: 0), CRP (baseline: 2 mg/L; week 24: 1 mg/L) and FC (baseline: 76 μg/g; week 24: 50 μg/g) were comparable between baseline and week 24 (Figure 3). Subgroups analysis of effectiveness based on the number of IFX switches showed similar findings (Figure 2). Sixteen patients required corticosteroids during follow‐up (one switch: 7/92, 7.6%; two switches 7/138, 5.1%; three switches: 2/62, 3%; *p* = 0.43).

### Immunogenicity and pharmacokinetics

At baseline IFX antibodies were detected in 33/276 patients (12%; one switch: *n* = 11 [4%], two switches *n* = 17 [6.1%], three switches: *n* = 5 [1.8%]; *p* = 0.56) of whom 10 (3.6%) discontinued IFX during follow‐up. Of 23 patients who remained on IFX, 14 (7.1%) patients had persistent antibodies during follow‐up whereas 9 (39.1%) patients had a transient antibody response.

De novo IFX antibodies were detected in 14 (7.1%) patients leading to IFX discontinuation in 5 (2.6%) patients. No differences were found in de novo IFX antibody development between subgroups based on number of switches (one switch: *n* = 7 [3.4%]; two switches: *n* = 7 [3.4%]; three switches: *n* = 0; *p* = 0.09).

IFX levels did not differ across different time‐points (baseline: 7.3 UI/ml; week 12: 6.5 UI/ml; week 24: 7.8 UI/ml, *p* = 0.06; Figure 3), although it should be taken into account that dose adjustments were allowed. Patients with fewer IFX biosimilar switches underwent dose intensification more frequently (one switch: 20/92 [21.7%], two switches: 14/138 [10.1%], three switches 4/67 [6%] *p* = 0.006). Four (1.3%) patients were moved to the subcutaneous IFX formulation during follow‐up.

### Safety

Six adverse events were reported in five patients. Adverse events included psoriatic reaction, squamous cell carcinoma of the tonsil, arthralgia, severe COVID infection requiring ICU hospitalisation, heart failure requiring ICU admission, and mild skin reaction. Of these adverse events, three were classified as severe adverse events leading to drug discontinuation.

## DISCUSSION

This is the first study evaluating the efficacy and safety of three successive switches in IBD patients treated with IFX. In a large real‐world cohort we showed that this approach is safe and effective with similar clinical and biochemical remission rates over time, irrespective of the number of switches.

Data on multiple biosimilar switches are scarce with most studies focussed on single switch and only three documenting double switch outcomes. There are no randomised control trials on IBD testing a multi switch approach. Single switch from IFX originator to CT‐P13 is recognised as safe and effective in IBD patients as demonstrated in clinical trials.^5^, ^6^ For double switches the data comes from observational cohorts^13^, ^20^, ^21^ and no data on IBD an triple switch has been reported yet. For this reason clinicians have relied on real‐world cohort studies despite limitations. Our group previously published the experience of the first biosimilar switch from originator to CT‐P13.^14^ This new real‐world IBD cohort provides effectiveness and safety data to support multiple switch approach.

The efficacy of double switch was recently published in a prospective multicentre study (*n* = 176 patients) comparing a single switch with a double switch group. Twelve months IFX persistence was 87% and better IFX persistence was found in the double switch group.^13^ These findings might be the result of selection bias since the double switch patients had a relatively long IFX duration, which is associated with a relatively lower IFX loss of response rate and a different immunogenicity profile.^22^ Indeed, we identified IFX duration rather than the number of IFX biosimilar switches as an independent predictor for IFX persistence. IFX duration and number of switches are correlated. This may explain their finding of better IFX persistence in the double switch group since they did not adjust for IFX duration. Furthermore, biochemical remission (CRP <5 mg/dl) at switch was independently associated with better IFX persistence in our study, rather than clinical or faecal biomarker remission (FC <250 μg/g). This may be explained by the known disconnect between symptoms and active inflammation in IBD and by collinearity in the multivariable model.^16^, ^23^

Multiple switch outcomes were investigated by the Sicilian Network for IBD in a prospective study with 276 patients, of which 192 patients started IFX de novo, 60 patients underwent a single IFX switch, and 24 had two IFX switches.^20^ No differences were observed in safety or drug persistency across the different groups with drug persistency at 48 weeks of 82.4%. These results are aligned with our findings with drug persistency at 90.6% at 7.5 months.

Further evidence that supports a comparable effectiveness of double switching from originator to CT‐P13 to SB2, compared to a single switch comes from a study involving 158 patients, 115 of whom had two switches and 43 a single switch.^21^ All patients were at the moment of switch in sustained steroid‐free clinical remission for at least 6 months. IFX persistence was 94.9% after median 54 weeks of follow‐up, which is slightly better compared to our findings (7.5‐month IFX persistence: 90.6%). This might be explained by the inclusion of only patients in sustained steroid‐free clinical remission. Indeed, clinical remission at switch was independently associated with better drug persistence in previous studies.^13^

Real‐world studies regarding adalimumab biosimilars have reported similar rates of effectiveness and safety in patients with one switch or two switches.^24^, ^25^ A phase III trial in psoriasis demonstrated no differences between patients who underwent four adalimumab biosimilar switches versus those who underwent none.^26^

Immunogenicity has been a major concern regarding multiple switches, although both our study and previous literature demonstrated that this seemed to be not happening more often to patients who had multiple switches compared to those who had less number of switches or none. Our study found 14 (7.1%) patients who developed de novo antibodies; none of them underwent three switches. This triple switch group may represent a selected cohort of patients on relatively long term IFX with a low immunogenicity risk.^27^ A previous French study observed that antidrug antibody formation was similar in patients with one switch or two IFX biosimilar switches.^28^ Moreover, IFX levels remained stable after switching.^14^, ^21^

Our study has several strengths including its prospective nature and the large sample size. Furthermore our study provides data for patients with a single switch, double switch and a triple switches. The prospective registrations of IFX start and stop dates, clinical scores, biochemical parameters and therapeutic drug monitoring contributes to completeness of the data by limiting selection bias during the collection of follow‐up data.

Nonetheless there are some limitations to our study. First the study design did not include a control arm that continued SB2, impeding the comparison of effectiveness and safety between groups. Although we were able to compare subgroups based on number of IFX switches, baseline characteristics were not comparable between subgroups (i.e. different IFX duration). Therefore we performed a multivariable analyses adjusting for these characteristics to assess IFX persistence. Second there were some missing data, although data were prospectively collected. This may have resulted in a conservative estimate of effectiveness outcomes since last observation was carried forwards for patients who discontinued IFX whereas missing data were censored for those who continued IFX. Third treatment optimisation was not standardised, but was performed at the discretion of the clinician. This reflects real‐world practice, allowing direct translation of results into clinical practice but this may have impacted outcomes. Of note, scrutiny of data collection during this study may have resulted into earlier dose adjustments and IFX discontinuation. Finally the cohort was heterogeneous in terms of disease activity, IFX doses and combination therapy.

## CONCLUSION

Multiple successive switches from the IFX originator to biosimilars appear effective and safe, irrespective of the number of switches. These findings are of major socioeconomic importance, especially in low and middle‐income countries where the access to healthcare may be limited.

## AUTHOR CONTRIBUTIONS

Beatriz Gros, Lauranne A. A. P. Derikx, Charlie W. Lees contributed to the design of the study. Beatriz Gros, Lauranne A. A. P. Derikx, Nikolas Plevris, Mathew Lyons, Claire O’Hare, Mathew Lyons, Colin Noble, Ian D. Arnott contributed to the data collection. Lauranne A. A. P. Derikx, Nikolas Plevris, Beatriz Gros, Nathan Constantine‐Cooke, Charlie W. Lees analysed the data. Beatriz Gros drafted the first version of the manuscript. All authors critically revised the manuscript for important intellectual content. All authors have approved the final version of this manuscript.

## CONFLICT OF INTEREST

Beatriz Gros has served as a speaker for Abbvie, Jansen, You&us and Galapagos. Nikolas Plevris has served as a speaker for Janssen, Takeda and Pfizer. Professor Charlie Lees has acted as a consultant to Abbvie, Janssen, Takeda, Pfizer, Galapagos, Bristol Myers Squibb, B.I., Sandoz, Novartis, GSK, Gilead, Vifor Pharma, Dr Falk, Trellus Health and Iterative Scopes; he has received speaking fees and travel support from Pfizer, Janssen, Abbvie, Galapagos, MSD, Takeda, Shire, Ferring, Hospira, and Dr Falk. Gareth‐Rhys Jones has served as a speaker for Takeda, Janssen, Abbvie and Ferring. Lauranne Derikx has served on advisory board for Sandoz and as a speaker for Janssen. Colin Noble has acted as a consultant to Galapagos. None of the other authors reported any conflicts of interest.

## Data Availability

All data are incorporated into the article and its online supplementary material.
